# Practices in urethral stricture management with drug-coated balloon dilatation: an international survey

**DOI:** 10.1007/s00345-026-06343-y

**Published:** 2026-04-07

**Authors:** Diarmuid D. Sugrue, John O’Connor, Łukasz Białek, Francesco Chierigo, Mikołaj Frankiewicz, François Xavier Madec, Behzad Abbasi, Leonidas Karapanos, Jakob Klemm, Mattia Lo Re, Juan Diego Tinajero, Jordán Scherñuk, Guglielmo Mantica, Paul Neuville, Maciej Oszczudłowski, Wesley Verla, Malte W. Vetterlein, Niall F Davis, Felix Campos-Juanatey, Elaine J. Redmond

**Affiliations:** 1 Department of Urology, Cork University Hospital, Cork, Ireland; 2 Strategic Academic Recruitment (StAR) Programme, Royal College of Surgeons in Ireland, Dublin, Ireland; 3 Department of Urology, Centre for Postgraduate Medical Education, Warsaw, Poland; 4 Department of Urology, Department of Health Science, University of Milan, ASST Santi Paolo e Carlo, Milan, Italy; 5 Department of Urology, Medical University of Gdańsk, Gdansk, Poland; 6 Department of Urology, Ambroise Paré–Hartmann Private Hospital Group, Paris, France; 7 Department of Urology, University of California, San Francisco, CA USA; 8 Department of Urology, Klinikum Leverkusen, Leverkusen, Germany; 9 Department of Urology, University Medical Center Hamburg-Eppendorf, Hamburg, Germany; 10 Department of Experimental and Clinical Medicine, University of Florence, Florence, Italy; 11 Unit of Oncologic Minimally Invasive Urology and Andrology, University of Florence, Careggi Hospital, Florence, 50100 Italy; 12 Chelsea Centre for Gender Surgery. Chelsea and Westminster Hospital NHS Trust, London, UK; 13 Department of Urology, Hospital Italiano de Buenos Aires, Ciudad Autónoma de Buenos Aires, Argentina; 14 Department of Surgical and Diagnostic Integrated Sciences (DISC), University of Genoa, Genoa, Italy; 15 Department of Urology, Hospital Lyon Sud, Hospices Civils de Lyon, Lyon, France; 16 Department of Urology, Ghent University Hospital, Ghent, Belgium; 17 Department of Urology, Beaumont Hospital, Dublin, Ireland; 18 School of Medicine, Unit of Andrology and Reconstructive Urology, Marqués de Valdecilla University Hospital, IDIVAL, Cantabria University, Santander, Spain; 19 School of Medicine, University College Cork, Cork, Ireland

**Keywords:** Urethral stricture, drug-coated balloon dilatation, urethroplasty

## Abstract

**Purpose:**

Drug-coated balloon (DCB) urethral dilatation which offers an alternative to standard endoscopic treatments of male anterior urethral stricture disease (AUSD). Its ease of delivery has facilitated its use by urologists with various subspecialty interests. The objective of this study was to characterise real-world practice patterns of a DCB device.

**Methods:**

An exploratory cross-sectional online survey was distributed to Optilume^®^ users via national and international urological societies and device distributor mailing lists. Descriptive and inferential statistics were performed using SPSS software.

**Results:**

*N* = 102 urologists responded to the survey of whom *n* = 47 (46%) were reconstructive subspecialists. DCB dilatation was predominantly performed under general anaesthesia (*n* = 59, 58%). Significant variation was seen with catheter duration, perioperative antibiotic use and post-procedure contraception advice. Off-label use was common with respondents offering DCB for penile urethral strictures (65%), primary treatment (64%) and bladder neck stenoses (65%). Higher-volume users (≥ 10/year) were more likely to perform DCB under flexible cystoscopy (OR 5.14, 95% 1.57–16.79, *p* = 0.007), bladder neck stricture (OR 4.66, 95% CI 1.55–14.03, *p* = 0.006), and for recurrences (OR 6.92, 95% CI 2.22–21.6, *p* = 0.001). Limited practitioner experience, an evidence gap, and the importance of shared decision making were highlighted on thematic analysis.

**Conclusions:**

This study provides an insight into the early experience a novel DCB among practicing urologists. Further research is required to optimize patient selection, procedural protocols and the understanding of long-term outcomes.

## Introduction

Male anterior urethral stricture disease (AUSD) is a challenging condition characterised by urethral fibrosis and cicatrisation leading to urinary outflow obstruction. It has an estimated incidence of 229–627 per 100,000 men [1]. Recurrence is common with conventional endoscopic treatments. Patency rates at 12 months post direct vision internal urethrotomy (DVIU) vary considerably from 8% − 77% [2–4] while recurrence in coaxial dilatation is between 24% and 65% [5–8]. Urethroplasty offers superior durability to dilatation yet requires subspecialist expertise and carries a greater morbidity.

Stricture dilatation using a drug-coated balloon (DCB) is a minimally invasive alternative to repeated endoscopic dilatation or open reconstruction. The Optilume^®^ device is a 0.038-inch over-the-wire catheter featuring a semi-compliant balloon coated with paclitaxel at 3.5 µg/mm^2^. It was United States Food and Drug Administration (FDA)-approved in 2021 [9, 10]. Paclitaxel is an antiproliferative chemotherapeutic which inhibits fibroblast proliferation and thus scar re-formation, thereby extending stricture-free intervals. ROBUST III was a randomized controlled trial including men with short (< 3 cm) AUSD and at least two prior failed endoscopic treatments, who were randomized to treatment with a DCB or standard of care (dilation or DVIU) [11]. The trial demonstrated higher anatomic patency rates at 6 months with DCB compared with standard of care (75% vs. 27%) and greater freedom from reintervention at 1 year (83% vs. 22%). Both ROBUST I (5-year follow-up) and ROBUST III (3-year follow-up) demonstrate durable long-term efficacy of DCB dilatation with freedom from repeat intervention rates of 72% and 71%, respectively [12]. Sustained symptom relief (IPSS reductions > 50%), improved Qmax (> 15 mL/s increase), and low PVR persisted without serious adverse events in both studies, supporting DCB as a viable alternative to urethroplasty in this setting [12, 13].

The straightforward deployment of a DCB has given reconstructive and general urologists alike an effective minimally invasive alternative to standard endoscopic treatment. However real-world utilisation patterns are as yet unknown. The current study therefore sought to characterise global practice trends among DCB practitioners, including procedural techniques, peri-operative management and post-treatment counselling. By delineating how a DCB is being used, we aim to inform evidence-based guidelines and identify targets for future clinical research.

## Methods

This study employed a targeted cross-sectional survey to identify real-world usage patterns for the DCB among practicing urologists worldwide. An exploratory approach was selected given the limited published data on contemporary DCB utilization practices. This design also allowed comprehensive investigation of previously undefined practice patterns and treatment preferences.

Survey domains were identified through a comprehensive background literature search, real-world clinical experience, and informal discussions among members of the European Association of Urology (EAU) Young Academic Urologists – Trauma and Reconstructive Urology Working Party. The identified topic areas included practitioner demographics, urethroplasty and reconstructive urology experience, DCB-specific experience and practice patterns, indications for device use, and postoperative care protocols.

An online questionnaire was distributed using *SurveyMonkey®*, a validated web-based survey platform (SurveyMonkey Inc, San Mateo, CA). The survey incorporated multiple-choice questions and Likert scales for standardized measurements, complemented by scoping free-text options to capture open-ended responses and qualitative insights. The full survey is included in the *supplementary materials*. Items were not randomized. Respondents were able to review and change their answers before submission. Completeness checks were carried out prior to distribution by user validation within the author group. Duplicate entries were avoided by assigning unique user identifiers based on cookies used by SurveyMonkey; the first entry was kept for analysis. Incomplete questionnaires were included in the analysis. No cut-off time was employed for completing the survey. Participant consent was obtained and responses anonymised.

The initial survey instrument underwent rigorous pilot testing among urologists and residents within the lead authors’ institutional units to ensure clarity, relevance, and overall user experience. Questions were modified to eliminate ambiguous terminology, enhance comprehension and ensure appropriate response options that were mutually exclusive. The survey underwent formal validity assessment. Face validity was established through expert review. Content validity was obtained through review by experienced members of EAU Young Academic Urologists – Trauma and Reconstructive Urology Working Party, who perform DCB procedures. The CHERRIES checklist was employed for web-based surveys (supplementary material) [14].

The target population comprised practicing urologists using DCB technology for management of male AUSD. Participants were invited with a purposeful sampling approach using a multi-channel dissemination strategy to reach reconstructive urologists internationally. The survey was initially distributed via specialty society mailing lists of the EAU Young Academics Urologists and Society of Genitourinary Reconstructive Surgeons (GURS) members, with onward forwarding by key opinion leaders to national and regional society mailing lists. In addition, the survey was distributed via Laborie’s Optilume™ urology contact database. Residents and fellows were not invited. Responses were voluntary and no incentives were provided. The survey was distributed in September 2024 and kept open for responses for three months. A multi-channel approach was used to maximize reach and ensure representation from diverse geographic regions and practice settings.

Descriptive analyses were conducted for demographic and practice characteristic. Preliminary inferential statistics were calculated for all variables to determine the association between respondent characteristics and DCB use. Non-parametric testing (Pearson’s χ²) was applied for categorical variables a priori given non-normal population distribution. Where > 25% of expected cell counts were *n* < 5 a Fisher’s exact test was substituted for 2 × 2 tables. All tests were two-tailed. A P-value < 0.05 was considered statistically significant. Statistical analyses were performed using IBM *SPSS Statistics (Version 31)* (IBM Corp., Armonk, NY).

Internal consistency of our instrument was assessed using Cronbach’s alpha coefficient. Thematic analysis of free text responses was undertaken using *Nvivo 14* (Lumivero, Denver, CO). The study was deemed exempt from ethical approval.

## Results

Responses were received from *N* = 102 urologists in 14 countries (Table 1). For the Laborie email distribution, the survey was delivered to 3,926 urology contacts, with 262 opens (6.7%) and 30 link clicks. Respondents were predominantly from the United States (*n* = 53, 52%), with significant representation from Ireland (*n* = 12, 12%) and Spain (*n* = 13, 13%). The remaining participants (*n* = 24, 24%) represented 11 other high-income countries across Europe and North America. Reconstructive urologists comprised the largest group (*n* = 47, 46%), followed by general urologists (*n* = 22, 22%). Subspeciality fellowship training was completed by the majority of respondents (*n* = 48, 67%). Career stage analysis showed an even distribution of experience (Table 1). Primary practice setting was predominantly academic (*n* = 63, 62%). Most participants were relatively new DCB users, with *n* = 63 (62%) using the device for 1–3 years and *n* = 33 (33%) using it for < 1 year. Over two-thirds of respondents performed less than 10 cases in the last 12 months (*n* = 68, 67%). DCB was reimbursed via insurance for the majority of users (*n* = 56, 55%).

**Table 1:** Characteristics Of Respondants

Total	*n* (%)
	102 (100)
Country	
USA	53 (52)
Ireland	12 (12)
Spain	13 (13)
Italy	4 (4)
France	8 (8)
UK	2 (2)
Norway	2 (2)
Canada	1 (1)
Poland	1 (1)
Portugal	1 (1)
Belgium	2 (2)
Turkey	1 (1)
Greece	1 (1)
Germany	1 (1)
Subspecialty	
Reconstructive	47 (46)
General	22 (22)
Female & Functional	10 (10)
Andrology	8 (8)
Endourology	8 (8)
Oncology	7 (7)
Fellowship Training	
Yes	68 (67)
Years Post Training	
≤ 9	49 (48)
10–19	31 (30)
≥ 20	22 (22)
Primary Practice Location	
Academic	63 (62)
Private Practice	25 (25)
Community & VA	14 (14)
Perform Urethroplasty	
Yes	66 (65)
Years Using DCB	
< 1	33 (33)
1–3	63 (62)
> 3	6 (6)
No. Performed Last 12 Months	
≤ 9	68 (67)
10–19	22 (22)
≥ 20	12 (12)
Funding	
Insurance	59 (58)
Public	33 (33)
Self Pay	10 (10)

*DCB* Drug coated balloon dilataion, *VA* veterans association

Table 2 outlines practice characteristics for the DCB. The procedure was performed predominantly under general anaesthesia (*n* = 59, 58%) or sedation (*n* = 32, 31%). Only nine respondents had ever used the DCB under local anaesthetic; users with ≥ 20 procedures per year were more likely to have performed DCB dilatation under local anaesthetic (odds ratio (OR) = 7.7; 95% confidence interval (CI)=[1.7–35], *p* = 0.014). Less than half of respondents performed a urethrogram at the time of procedure (*n* = 45, 44%). The majority predilated the target stricture prior to DCB placement (*n* = 71, 70%). The DCB was predominantly placed with a rigid cystoscope (*n* = 58, 57%). Median catheter duration was three days (IQR 2–4).

**Table 2:** Practice Characteristics Of RespondentsTable 2

Total	*n* (%)
	99 (100)
Anaesthetic Use	
General Anaesthesia	59 (58)
Sedation	32 (31)
Regional	7 (7)
Local	1 (1)
MSU ≥ 2 days prior	
Yes	78 (78)
Antibiotic Use	
Single dose	67 (66)
Post-op Course	28 (28)
None	4 (4)
Peri-Op Urethrogram	
Yes	45 (44)
Predilate Stricture	
Yes	71 (70)
Balloon Diameter	
18ch	7 (7)
24ch	30 (30)
30ch	62 (61)
DCB Placement	
Rigid cystoscope	58 (59)
Flexible cystoscope	18 (18)
Fluoroscopy only	22 (22)
Catheter Size	
12Ch	7 (7)
14Ch	36 (36)
16Ch	52 (51)
18Ch	2 (2)
No catheter	2 (2)
Catheter Duration (Days)	
Median (IQR)	3 (2–4)
Off-Label Use (*n* = 94)	
Long Stricture (> 3 cm)	42 (45)
Bladder neck stricture	61 (65)
Penile stricture	66 (70)
Primary treatment	65 (69)
Post urethroplasty	62 (66)
Recurrent stricture	50 (53)

*DCB* drug coated balloon, *IQR* interquartile range, *MSU* mid stream urine

Significant variability was seen in contraceptive counselling following DCB placement. Barrier contraception was the most commonly recommended *n* = 52 (51%) followed by abstinence (*n* = 27, 27%). Some practitioners (*n* = 12, 12%) provided no contraceptive advice. Only *n* = 44 (43%) of urologists recommended barrier contraception or abstinence for 30 days, in line with manufacturer recommendations, while *n* = 22 (22%) advising longer times. Median duration of conception avoidance with female partners of child-bearing age was 19 weeks (IQR 12–26). Notably, *n* = 27 (27%) provided no specific conception advice.

Approximately two-thirds of respondents would offer a DCB dilatation in penile urethral strictures (*n* = 66, 65%), or for primary stricture treatment (*n* = 65, 64%). The majority would offer a DCB off-label to patients with bladder neck stenoses (*n* = 66, 65%), long strictures >3 cm (*n* = 42, 45%), or in post-urethroplasty recurrence (*n* = 62, 61%). Three participants did not respond to questions about their DCB practice and seven declined to answer regarding off-label usage. Cronbach’s α for scale items regarding surgeon experience was 0.51, indicating acceptable internal consistency of our instrument.

Thematic analysis of DCB use in various clinical scenarios revealed several patterns. Firstly, limited practitioner experience and an evidence gap were noted. For penile urethral strictures, respondents emphasized a lack of robust evidence, with comments like “Lack of data,” “Not confident of this as yet,” and “Waiting for further data.” However, cautious optimism was seen in bladder neck stenosis with several reporting “good results” “good short-term success,” or “very effective” in highly selected patient groups such as post-prostatectomy, albeit with limited experience. Secondly, urethroplasty as the gold standard in male AUSD was highlighted. For long (> 3 cm) strictures, “urethroplasty is a better option” appeared repeatedly. With post-urethroplasty stricture recurrence, mixed success was reported using DCB, with a mix of “this has been successful,” “used < 5 times with < 50% success,” and “I perform formal graft urethroplasty” as responses.

Thirdly, practitioners acknowledged comorbidities and shared decision making as influencing treatment choice. Some may use DCB up to 4 cm for “patients who are poor candidates for urethroplasty”, “elderly, unfit” or where the choice was “patient driven”. DCB was seen as an alternative when conventional options were declined, for patients “not willing to undergo reconstruction” or “wanting to avoid urethroplasty.” Lastly, cost constraints and billing influence practice. Regarding performing DCB under local anaesthetic, in the US “Billing…requires that it’s done in an operating room.” For primary treatment of AUSD, some “offer to try dilation first due to cost” or noted “can’t get authorization for this.

## Discussion

Paclitaxel-coated balloon catheter is a novel treatment for male AUSD. We conducted a scoping analysis of real-world DCB use among a purposeful sample of international urologists. Our study demonstrates that the majority of practitioners are early in their use of DCB with limited but growing experience. While reconstructive urologists predominated in the DCB’s uptake, a significant number of non-reconstructive subspecialists who do not perform urethroplasty are utilising the technology, alluding to the ease of use and shorter learning curve compared to open urethroplasty [15].

The use of a DCB under local anaesthesia has been described in small cohorts with a high degree of tolerability and patient satisfaction [16]. Almost all our respondents performed DCB dilatation in an operating room setting with anaesthetic support. Whilst this may be influenced by reimbursement patterns, particularly in insurance-based health systems, it suggests that there is a preference for general anaesthesia early in the learning curve. As such, there is significant scope to assess the safety and efficacy of a DCB under local anaesthetic and in the office setting.

Significant variability in practice patterns were seen across our cohort. Although recommended by the manufacturer, the majority of respondents do not perform a peri-operative urethrogram, while a large minority do not predilate the stricture [17]. Median catheter duration was three days with the majority using a 14–16 F Foley. More outcome data is required to assess the effect these parameters may have on patient satisfaction and stricture recurrence. While the EAU offers no specific guidance on perioperative antibiotics for standard urethral dilatation or DCB [18], the American Urological Association (AUA) advise preoperative urine cultures and single dose antibiotic use during AUSD treatment [19] which our respondents predominantly adhered to.

The EAU weakly recommends DCB for short (< 3 cm) bulbar strictures recurring after at least two prior endoscopic treatments, but only in patients for whom urethroplasty is not an option [20]. Meanwhile, the AUA offer a conditional (Grade B) recommendation for DCB in combination with urethral dilation or DVIU, in recurrent bulbar urethral strictures < 3 cm regardless of previous treatments [19]. The high degree of acceptance of DCB as a primary treatment in our cohort (64%) suggests it is not considered merely as a salvage therapy. Our study demonstrates that many practitioners are willing to offer a DCB in off label scenarios including long strictures or bladder neck stenoses, or outside international guidance including penile stricture and post-urethroplasty. While DCB has been described for such scenarios in small cohorts and case reports, given the apparent pervasiveness and willingness for off label use among urologists, robust outcome data is a required as a matter of urgency [15, 21–23].

The paclitaxel payload of the DCB raises concerns about systemic toxicity and partner transmission. Reassuringly, large registry data have not observed an excess mortality with paclitaxel-coated vascular devices [24]. However, semen accumulation of paclitaxel does occur with DCB; pharmacokinetic studies have demonstrated detectable paclitaxel in approximately one-third of patients at 6 months post-procedure [17, 25]. The Instructions for Use in the United States advise abstinence or barrier contraception for 30 days post-procedure and avoid fathering a child for ≥ 6 months [17]. In Europe, the product manual advises patients to undertake protected intercourse (condom) for 30 days post procedure and those with partners of childbearing potential should use a condom for at least 90 days post-treatment [10]. This guidance discrepancy may in part explain the divergent post-procedure advice given by practitioners in our survey. However, it is possible that others are simply not aware that post-procedure contraception advice is required, as demonstrated by a notable proportion of respondents who offered no advice to patients.

Our study demonstrates that practice variation in DCB deployment is driven more by practical experience and subspecialty expertise than practice setting or funding model. Experienced users were more likely to use flexible cystoscopy or consider performing DCB dilatation under local anaesthesia, suggesting an early learning curve. Non-reconstructive specialists were more likely to offer a DCB for primary AUSD treatment, suggesting a shifting paradigm for stricture management for the non-reconstructive or general urologists.

Our thematic analysis of free-text responses highlights the main sentiments, concerns and conditional approaches among practitioners. DCB is considered a valid alternative but inferior to urethroplasty; this highlights the importance of shared-decision making and appropriate counselling of patients. Overall, practitioners appeared reservedly hopeful for the DCB’s efficacy but currently operate in a knowledge gap. Robust evidence is required to inform optimum perioperative antibiotic use, catheter size and duration, and contraception advice as demonstrated by the wide variation in responses in our cohort.

Several methodological limitations are acknowledged in this study design. First, the outcomes of AUSD treatment are influenced by a complex interplay of patient factors (such as age, comorbidities, and smoking status), stricture characteristics (including length, location, aetiology, and prior interventions), and surgical factors (such as procedure and technique, operator experience, and adherence to protocols). This survey did not capture detailed patient-level data or stricture-specific variables, nor did it assess practitioner technical variations beyond basic demographics, limiting our ability to correlate practice patterns with real-world efficacy. Secondly, the purposeful sampling approach, while appropriate for targeting specialized practitioners, may introduce selection bias towards early adopters and enthusiasts. Responders were predominantly from high-income countries in Europe and North America; these selection biases may limit generalizability, particularly in the context of a small sample population. The cross-sectional design captures practices at a single time point, potentially missing temporal variations in usage patterns. Because invitations were forwarded through multiple external mailing lists a single denominator could not be enumerated; therefore, an overall response rate could not be calculated, raising the possibility of selection and nonresponse bias. Thirdly, the reliance on self-reported data introduces the possibility of recall bias, social desirability bias, or non-response bias. However, the internet-based format facilitated international participation and accessibility and allowed for comparison of practice patterns. Our survey was validated by members of the EAU Young Academic Urologists – Trauma and Reconstructive Urology Working Party with an acceptable internal consistency. Despite these limitations, this scoping study provides a platform from which to undertake further research into DCB use as it becomes more prevalent.

## Conclusion

There appears to be a wide variation in how DCBs are used in male AUSD with off-label use common. These findings underscore the need for evidence-based guidelines, prospective registries, and long-term outcome studies to optimize patient selection and standardize procedural protocols.

## Data Availability

Raw data available on request.
